# Supplemental Protein during Heavy Cycling Training and Recovery Impacts Skeletal Muscle and Heart Rate Responses but Not Performance

**DOI:** 10.3390/nu8090550

**Published:** 2016-09-07

**Authors:** Andrew C. D’Lugos, Nicholas D. Luden, Justin M. Faller, Jeremy D. Akers, Alec I. McKenzie, Michael J. Saunders

**Affiliations:** Department of Kinesiology, James Madison University, 261 Bluestone Drive MSC 2302, Harrisonburg, VA 22807, USA; dlugosac@dukes.jmu.edu (A.C.D.); ludennd@jmu.edu (N.D.L.); fallerjm@dukes.jmu.edu (J.M.F.); akersjd@jmu.edu (J.D.A.); mckenzai@dukes.jmu.edu (A.I.M.)

**Keywords:** carbohydrate, protein, chocolate milk, muscle repair, sports nutrition

## Abstract

The effects of protein supplementation on cycling performance, skeletal muscle function, and heart rate responses to exercise were examined following intensified (ICT) and reduced-volume training (RVT). Seven cyclists performed consecutive periods of normal training (NT), ICT (10 days; average training duration 220% of NT), and RVT (10 days; training duration 66% of NT). In a crossover design, subjects consumed supplemental carbohydrate (CHO) or an equal amount of carbohydrate with added protein (CP) during and following each exercise session (CP = +0.94 g/kg/day protein during ICT; +0.39 g/kg/day during RVT). A 30-kilometer time trial performance (following 120 min at 50% W_max_) was modestly impaired following ICT (+2.4 ± 6.4% versus NT) and returned to baseline levels following RVT (−0.7 ± 4.5% versus NT), with similar responses between CHO and CP. Skeletal muscle torque at 120 deg/s benefited from CP, compared to CHO, following ICT. However, this effect was no longer present at RVT. Following ICT, muscle fiber cross-sectional area was increased with CP, while there were no clear changes with CHO. Reductions in constant-load heart rates (at 50% W_max_) following RVT were *likely* greater with CP than CHO (−9 ± 9 bpm). Overall it appears that CP supplementation impacted skeletal muscle and heart rate responses during a period of heavy training and recovery, but this did not result in meaningful changes in time trial performance.

## 1. Introduction

Athletes perform condensed periods of overload training to elicit compensatory adaptations and performance gains. These planned periods of heavy training often lead to transient decrements in physiological function and performance capacity [1,2,3]. Numerous reports suggest that co-ingestion of carbohydrate and protein (CP) proximal to a single session of heavy exercise can enhance subsequent performance compared to carbohydrate (CHO) treatments matched for carbohydrate content [4], or total calories [5,6,7,8]. CP supplementation may therefore be a feasible strategy to promote recovery, minimize performance decrements, and maximize compensatory adaptations during periods of heavy training and recovery. However, comparatively few studies have assessed the efficacy of CP supplementation over multiple days of training, and although there are some reports that CP can enhance subsequent performance under these conditions [9,10,11], the effects of CP on functional recovery across multiple days of training remains unclear [12,13,14].

An important feature shared by the aforementioned multi-day studies is that performance levels were sustained over multiple days of heavy training, even in the CHO control conditions. Very little is known about the value of CP supplementation during an extended period of rigorous training that leads to impaired physical function and performance. One related study examined the effects of a protein-enriched diet (3.0 vs. 1.5 g/kg/day) during seven days of intensified training followed by seven days of reduced-volume training [15]. Higher protein intake resulted in ‘possible attenuation’ of performance decrements following the intensified training period, as well as a ‘possible benefit’ on performance restoration after the reduced-training period. By contrast, a recent study supplemented CP during exercise throughout six days of strenuous training that elicited decrements in performance, and reported no differences in performance versus an isocaloric CHO supplement (with CP provided post-exercise in both conditions) [16]. However, neither of these studies specifically examined the impact of CP when provided immediately post-exercise, which is potentially significant because relatively modest increases in protein intake provided post-exercise (i.e., 0.4 g/kg/h) have been shown to positively impact next-day exercise performance versus CHO [8]. Therefore, a primary purpose of the present investigation was to investigate the effects of CP provided during and post-exercise on exercise performance following multiple days of strenuous cycling training, and a subsequent period of recovery.

The ostensible benefit of CP supplementation during heavy periods of endurance training could be mediated through the effects of protein on skeletal muscle. There is evidence that skeletal muscle undergoes a markedly different global transcriptional response to endurance exercise when protein is added to a supplement containing carbohydrate and fat [17]. Among other pathways, gene expression related to immune and inflammatory processes and extracellular matrix and cytoskeletal remodeling appeared to be favorably influenced by the presence of exogenous protein. In support of these molecular alterations, CP supplementation close to acute heavy endurance exercise can better preserve subsequent whole muscle function and reduce indices of post-exercise muscle damage (i.e., muscle soreness and biomarkers of sarcolemma permeability) compared to CHO alone [18,19]. Several reports also indicate that CP can continue to attenuate markers of muscle damage across multiple days of exercise [9,12,20,21]. However, very little is known about how this may impact whole muscle function and performance during periods of heavy training and subsequent recovery. Thus, the present study was also designed to examine the effects of CP supplementation on changes in muscle fiber cross-sectional area (CSA), muscle function, and markers of muscle damage during consecutive periods of strenuous cycling training and recovery.

CP intake may also impact endurance performance via changes in cardiovascular responses during exercise. For instance, cycling performance was enhanced during the latter stages of an 8-day stage race in those consuming CP versus CHO alone [22]. The CP group also had attenuated changes in body temperature during exercise, and tended to have lower exercise heart rates, suggesting performance benefits were related to altered cardiovascular responses with CP. This view is strengthened by observations in untrained individuals that CP ingestion was associated with increased plasma albumin content and plasma volume expansion [23,24,25], resulting in increased stroke volume and decreased heart rate during exercise versus placebo [24,25]. So, there is emerging evidence that CP may influence cardiovascular responses during repeated days of heavy cycling, but these effects have not yet been systematically investigated in endurance athletes. Therefore, the present study also examined the effects of CP supplementation on heart rate responses during constant-load cycling following consecutive periods of strenuous cycling training and recovery.

## 2. Materials and Methods

### 2.1. Subjects

Ten endurance-trained cyclists from James Madison University and the Harrisonburg area volunteered for this study. All subjects were experienced cyclists who reported ≥7 h·week^−1^ of cycling training for ≥2 months prior to the investigation (including at least one ride ≥3 h every 14 days), and demonstrated VO_2peak_ values ≥50 mL·kg^−1^·min^−1^. One subject failed to complete the study due to time demands and another was unable to adhere to dietary and training controls. An additional subject completed all trials, but exhibited substantial variations in exercise performance between baseline trials conducted at the onset of each experimental condition (i.e., variability in time trial performance >3 SD larger than those from all other subjects). Thus, performance data for this subject was removed, and data are reported for 7 subjects (5 males, 2 females; age, 25 ± 8 year; height, 173 ± 12 cm; weight, 71 ± 12 kg; VO_2peak_, 63 ± 9 mL·kg^−1^·min^−1^). Subjects gave consent to participate after receiving written and oral information regarding experimental procedures and potential risks. All procedures were approved by James Madison University’s Institutional Review Board (Protocol #12-0487).

### 2.2. Preliminary Testing

An incremental-load cycling test was conducted on a computerized ergometer (Velotron, Racermate Inc., Seattle, WA, USA) to determine VO_2peak_, as described previously [26]. Oxygen uptake was assessed throughout the test using indirect calorimetry via an automated Moxus Modular Metabolic System (AEI Technologies, Bostrop, TX, USA). VO_2peak_ was recorded as the highest 30-s mean VO_2_ value, and was used to determine if subjects met the inclusion criteria. Power output at VO_2peak_ (W_max_) was used to prescribe workloads for subsequent testing.

### 2.3. Experimental Design

**Figure 1:**
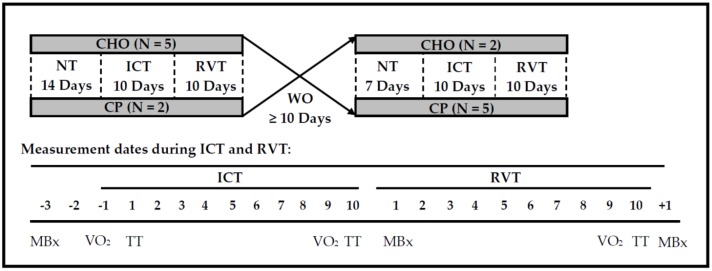
General Study Design. NT = normal training; ICT = intensified cycle training (daily training duration = ~220% NT); RVT = reduced volume training (~65% of NT); WO = washout; CHO = carbohydrate supplementation; CP = carbohydrate + protein supplementation; MBx = Muscle function and biopsy; VO_2_ = VO_2peak_; TT = Cycling time trial, constant-load exercise, and blood biomarkers.

The general study design is illustrated in Figure 1. Briefly, following a baseline period of normal training (NT), subjects completed two 20-day training blocks separated by a washout period (WO). Each training block was divided into 10 days of intensified cycling training (ICT) followed by 10 days of reduced volume training (RVT). Nutritional supplementation (CHO or CP) was provided throughout the ICT and RVT periods. A double-blind crossover design was utilized such that each subject received both nutrition interventions, with order of nutritional treatments randomly counterbalanced. Due to uneven subject retention, two subjects completed the CP trial first, and five subjects completed the CHO trial first. Details regarding training and nutritional interventions are provided below.

#### 2.3.1. Normal Training

The first 7 days of NT were used to quantify normal training volumes to prescribe training volumes and intensities for the remainder of the study. The second 7 days were used to conduct familiarization trials and preliminary testing for exercise protocols while maintaining total training duration and intensity at normal training levels. Subjects utilized bicycle wheels equipped with a PowerTap system (Saris Cycling Group Inc., Madison, WI, USA) to quantify power output, heart rate, cycling duration and distance during all training sessions conducted outside the laboratory. Power output and heart rate during cycling were used as indices of training intensity, whereas training duration (min) was used to quantify training volume.

#### 2.3.2. Intensified Cycling Training

ICT consisted of 10 days in which average daily training duration was increased to ~220% of NT levels. This overload was comparable to previous training protocols that resulted in impaired performance following ICT [2,27]. Preloaded time trials (120 min at 50% W_max_ + 30-km maximal effort, described below) and VO_2peak_ tests were completed on the days shown in Figure 1 (additional preloaded time trials were conducted on days 4 and 7 to contribute to total training loads). Additional training was conducted outside of the laboratory; subjects followed individualized training guidelines to achieve 220% of NT training duration. Training was quantified with a PowerTap system, as described above. During the second treatment phase, subjects were provided with specific durations and intensities to match the training stimulus with the first treatment phase.

#### 2.3.3. Reduced Volume Training

Average daily training duration was reduced to ~65% of NT levels during RVT, with the intent of restoring or improving cycling performance following ICT [15,28]. Preloaded 30-kilometer time trials and VO_2peak_ tests were completed on the days shown in Figure 1. Otherwise, all training during RVT was completed outside the laboratory, quantified as described above to verify compliance with the training guidelines. During the second treatment phase, subjects were provided with specific durations and intensities to match the training stimulus with the first treatment phase.

#### 2.3.4. Washout (WO) Period

WO consisted of individualized periods of recovery to accommodate schedules, with the intent of restoring NT loads prior to the second phase of the study. A minimum of 10 days of WO was provided, resulting in ≥27 days between the two periods of ICT (i.e., 10 days RVT + 10 days WO + 7 days NT). In female subjects, the washout period was timed to ensure that each treatment phase (CHO or CP) began on the same day of the menstrual cycle, to offset any potential influences of menstrual phase on study outcomes.

### 2.4. Treatment Beverages

Treatment beverages were administered during (750 mL·h^−1^) and immediately following (11.8 mL·kg·BW^−1^) all training sessions throughout ICT and RVT. Recovery beverages were consumed within 30 min of terminating exercise. Participants avoided any other beverage or food intake for 2 h following each exercise session, with the exception of ad libitum water consumption.

The treatment beverages consumed during exercise were either a commercially available carbohydrate-electrolyte beverage (CHO), providing 45 g·h^−1^ carbohydrate (sucrose/dextrose), 423 mg·h^−1^ sodium, and 97 mg·h^−1^ potassium (Gatorade^®^, PepsiCo, Inc., Purchase, NY, USA); or an identical beverage plus 17.7 g·h^−1^ of hydrolyzed whey protein isolate powder (American Casein Company, AMCO, Burlington, NJ, USA), providing 15 g Pro·h^−1^ (CP). The post-exercise treatment beverages were either a chocolate-flavored carbohydrate beverage (CHO), containing 1.2 g CHO kg·BW^−1^ (maltodextrin/sucrose), 0.09 g fat kg·BW^−1^, 3.3 mg·kg·BW^−1^ sodium, and 4.4 mg·kg·BW^−1^ potassium (Clif Shots, Clif Bar and Co., Emeryville, CA, USA); or commercially-available low-fat chocolate milk (TruMoo^®^, Dean Foods, El Paso, TX, USA), containing 1.2 g CHO·kg·BW^−1^ (lactose/sucrose), 0.4 g protein·kg·BW^−1^, 0.11 g fat·kg·BW^−1^, 9.0 mg·kg·BW^−1^ sodium, and 21.0 mg·kg·BW^−1^ potassium (CP).

### 2.5. Dietary Controls

Participants were provided detailed instructions on dietary procedures and recording techniques by a Registered Dietician Nutritionist (RD/RDN). All subjects were provided with a digital food scale for food measurement, and completed a food intake record (FIR) during NT (4 days) and ICT (10 days for each treatment) and RVT (10 days for each treatment). The FIR were analyzed using the Nutrition Data System for Research (University of Minnesota, Minneapolis, MN, USA) and evaluated by the RD/RDN for quality assurance. FIR from NT were analyzed to ensure that baseline carbohydrate intake was >6.5 g/kg/day (all subjects exceeded this level). The FIR obtained from the first treatment phase (ICT and RVT) were used to develop replicable menus for the second treatment phase, which included three alternative choices for each food item that matched calories and macronutrients. Participants were instructed to use these menus to replicate their dietary habits during the second phase of the crossover design. During this second treatment phase participants submitted their FIR each day by 4:00 p.m. and researchers analyzed dietary intakes immediately upon receipt. Researchers responded to participants via text message or e-mail with specific dietary prescriptions for the remainder of the day to ensure that daily calories and macronutrient intakes remained consistent with the previous treatment period.

During all exercise trials conducted in the laboratory, subjects received 250 mL of beverage every 20 min until exercise completion. For all rides performed outside the laboratory, participants received bottles of treatment beverage measured to achieve 750 mL/h of fluid ingestion for the duration of each training ride. Subjects returned empty bottles to the investigators (including any remaining solution, to ensure compliance) following each ride. Following each exercise session, participants received recovery beverages and were instructed to finish the beverage within 30 min of terminating exercise. Participants avoided any other beverage/food intake for 2 h following each exercise session, with the exception of ad libitum water consumption. During the time period between the onset of each training session and two hours following each training session, participants received no nutrients other than the CHO or CP treatment beverages. All laboratory testing was performed after an 8–10 h overnight fast (ad libitum water consumption). In addition, participants were provided with standardized boxed-lunches on all days they reported to the laboratory (8/20 days per treatment period), which standardized dietary intake for approximately 6 h after each exercise session.

### 2.6. Dependent Measurements

#### 2.6.1. Cycling Performance and Responses to Constant-Load Exercise

Cycling tests were conducted on a computerized cycle ergometer (VeloTron, Racermate Inc., Seattle, WA, USA) at the completion of NT, ICT and RVT (see Figure 1). Trials began with 120 min of cycling at 50% W_max_ (from VO_2peak_ test during NT of the first treatment phase). This provided a period of constant-load exercise in which physiological measurements (described below) could be compared between training periods and treatments at the same absolute intensity. It also provided a prolonged, metabolically demanding duration of exercise prior to the second portion of the trial; a self-paced, simulated 30-kilometer time trial (TT), which was used to quantify cycling performance. Measurements obtained during the cycling tests are described below:

##### Cardiorespiratory Responses to Constant-Load Exercise

Oxygen uptake (VO_2_) and respiratory exchange ratio (RER) were assessed using a Moxus Modular Metabolic System (AEI Technologies, Pittsburgh, PA, USA) at 25–30 min of constant-load cycling at 50% W_max_. Values were averaged over the final three min of data collection, following two minutes of breathing equilibration.

Heart rate (Suunto, Vaanta, Finland) and ratings of perceived exertion (RPE; 6–20 Borg Scale) were recorded at 30 min of cycling at 50% W_max_. Finger-stick blood samples (~0.5 mL) were obtained at the same time; glucose and lactate levels were determined immediately from whole blood using automated instrumentation (YSI 2300 STAT glucose/lactate analyzer, Yellow Springs, OH, USA).

##### Cycling Performance

The 30-kilometer TT finishing times and average power output were used as measures of cycling performance. Subjects were encouraged to treat the TT portion of each trial as a competitive event and provide a maximal effort. Subjects received no performance feedback during the TT other than elapsed distance, and no verbal encouragement was provided. A pedestal fan was placed ~2 m from the handlebars and utilized on high speed setting for consistency across trials. This protocol has been utilized previously in our laboratory with good reproducibility (coefficient of variation between repeated trials of 3.6%).

#### 2.6.2. VO_2peak_ and Body Weight

Subjects completed graded exercise tests to obtain VO_2peak_ values following NT, ICT and RVT (see Figure 1), using protocols described above. Body weight was measured prior to the onset of these trials, following an overnight fast and voiding of the bladder, and while wearing only cycling shorts (all subjects) and sports bra (females).

#### 2.6.3. Peak Isokinetic Torque

Torque of the knee extensors from a single, randomly selected leg was assessed using an isokinetic dynamometer (Biodex Medical System Inc., Shirley, NY, USA) following NT, ICT and RVT (Figure 1). Following 5 min of standardized warm-up on a cycle ergometer, peak isokinetic torque was assessed at 240 deg·s^−1^ and 120 deg·s^−1^. Four maximal trials were completed at each velocity, with the highest value recorded as the peak value. Trials were separated by 30 s of rest. Furthermore, opposite legs were used for peak torque assessments between CHO and CP training phases.

#### 2.6.4. Muscle Soreness

Subjects rated their perceived muscle soreness using a 100 mm visual analog scale, with 0 indicating no muscle soreness and 100 indicating extreme soreness, as described previously [13]. Scores were obtained at the end of each training period (Figure 1).

#### 2.6.5. Muscle Fiber Cross-Sectional Area

Muscle biopsies were obtained from the *vastus lateralis* (VL) following each training phase (Figure 1). Biopsy samples were processed, stored, and mounted onto microscope slides as previously described [29]. Briefly, slides were incubated in primary antibodies directed against laminin, myosin heavy chain (MHC) I and IIa (Developmental Studies Hybridoma Bank, Iowa City, IA, USA). Following a series of washes, slides were then incubated in appropriate fluorescently labeled secondary antibodies (Invitrogen, Molecular Probes, Carlsbad, CA, USA) and visualized on an upright fluorescent microscope (Nikon Eclipse TE2000-E, Tokyo, Japan). Fiber CSA was measured using ImageJ software (National Institutes of Health, Bethesda, MD, USA). Due to insufficient tissue yields for two subjects during RVT (with CP supplementation), this time point was excluded from statistical analysis.

#### 2.6.6. Biomarkers

Fasting venous blood samples were obtained from an antecubital vein following 10 min of supine rest following NT, ICT and RVT (prior to each TT). After 30 min of coagulation, samples were centrifuged at 3000 rpm for 10 min at 4 °C. Serum was separated and stored at −80 °C for later analysis.

Serum albumin was assessed via the bromocresol green method using spectrophotometry at an optical density of 620 nm, according to standardized procedures provided by the manufacturer (KA1612, Abnova, Walnut, CA, USA). Serum creatine kinase was analyzed by an enzymatic kinetic assay (Pointe Scientific, Canton, MI, USA) on an automated biochemical analyzer (ChemWell-T 4600, Awareness Technology Inc., Palm City, FL, USA). Serum cortisol concentrations were measured using a Quantikine^®^ high sensitivity enzyme-linked immunoassay (KGE008, R&D Systems, Minneapolis, MN, USA). The minimal detectable concentrations were 0.01 g·dL^−1^ for albumin, 1.0 U/L for creatine kinase, and 0.156 ng·mL^−1^ for cortisol.

#### 2.6.7. Statistical Analyses

For each measurement variable, magnitude-based inferences about the data were derived using methods described by Hopkins [30]. All data was log transformed to diminish the effects of non-uniformity. The threshold for the smallest meaningful treatment effect was quantified as 0.2*SD (obtained from NT in the CHO condition) for all variables other than cycling performance (see below). A published spreadsheet was used to determine the likelihoods of the true treatment effect (of the population) reaching the meaningful change threshold [31]. Likelihoods were classified as: <1% almost certainly no chance, 1%–5% = *very unlikely*, 5%–25% = *unlikely*, 25%–75% = *possible*, 75%–95% = *likely*, 95%–99% = *very likely*, and >99% = *most likely*. If the likelihood of the effect reaching the threshold was <25% and the effect was clear, it was classified as a ‘trivial’ effect. If 90% confidence intervals included values that exceeded the threshold for both a positive and negative effect, effects were classified as unclear.

As recommended by Paton and Hopkins [32], the smallest worthwhile change in performance was defined as 0.3 × the within-subject variation of competitive cyclists across repeated trials (CV = 1.3% for time and estimated 3.25% for power), which translated to an effect of 0.22 min or 2.0 watts in the current study. Clinical inference criteria were used from published spreadsheets to classify the effects of treatment on performance time [31]. The statistical power of our research design was calculated using a publicly available spreadsheet created for studies using magnitude-based inferences [33]. Based on a minimum sample size of seven subjects (and estimating within-subject variability based on prior studies in our laboratory using the same measurement protocol), the present design and statistical approach possessed the statistical power to detect changes in time trial performance of 3% (1.7 min) with a power of 0.9; effects on time trial performance of 2% (1.1 min) could be detected with an estimated power of 0.54.

For ease of interpretation, data are displayed as raw means ± SD and/or mean difference between treatments ± CL (90% confidence limit). Effects of treatment order were examined using the same statistical approach described above. No meaningful effects of treatment order were observed for any variables (i.e., order effects *unclear* or *trivial*), other than VO_2peak_ (*likely*) and skeletal muscle function measurements (Peak Torque 120 (NT vs. RVT) = *very likely*; Peak Torque 240 (ICT and NT vs. RVT) = *very likely*). Therefore, order-adjusted outcomes are reported where appropriate in addition to raw values.

## 3. Results

### 3.1. Training Loads and Nutrient Intake

Training duration, power output, and heart rate during NT, ICT, and RVT are reported in Table 1. These data were used to quantify compliance with the prescribed training program, and assess the consistency of training completed between the CHO and CP treatment phases. Average daily training duration during ICT was increased to 222% of NT levels (*most likely* increased versus NT in both CHO and CP). Duration during RVT was 66% of NT levels (*most likely* decreased versus NT in both CHO and CP). Average training intensity decreased slightly but consistently during ICT. Specifically, average power output and heart rates during ICT training were lower versus NT and RVT in both CHO and CP (all semantic inferences = *likely*/*very likely*/*most likely*). Thus, training during ICT represented a substantial increase in average daily training duration, at a slightly lower average exercise intensity. The RVT training represented a substantial decrease in training duration, at the same average intensity as NT. As intended, overall training demands between CHO and CP were very similar within each training period.

Macronutrient intake during ICT and RVT are reported in Table 2. Differences in macronutrient intake between CHO and CP are shown independent of treatment beverages (Dietary Macronutrients) and including the treatments (Total Macronutrient Intake). During ICT, dietary macronutrients were virtually identical between CHO and CP phases, and the increased protein/calories from the CP treatment beverages resulted in higher total protein/calories in CP. During RVT, subjects tended to consume slightly less dietary carbohydrate and fat (and thus calories) in CP. Due to the higher protein/calories from the CP treatment beverages, this resulted in similar total caloric intake between CHO and CP.

### 3.2. Dependent Measurements

#### 3.2.1. Cycling Time Trial (TT) Performance

**Figure 2:**
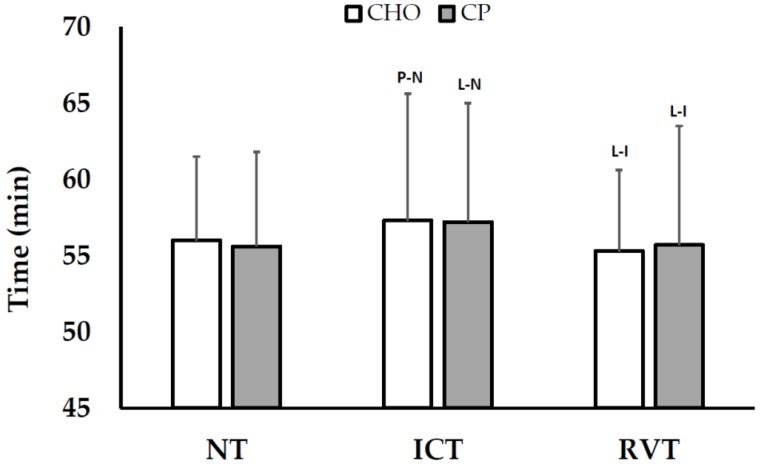
Cycling Performance (Mean ± SD) during Normal, Intensified, and Reduced-Volume Training. Within-treatment effects: P-N = *Possibly* different than NT; L-N = *Likely* different than NT; L-I = *Likely* different than ICT. No between-treatment effects were observed. NT = normal training; ICT = intensified cycle training; RVT = reduced volume training; CHO = carbohydrate supplementation; CP = carbohydrate + protein supplementation.

Pre-loaded 30-km TT times are shown in Figure 2, and tended to get slower from NT (CHO: 56.0 ± 5.3 min; CP: 55.6 ± 6.2 min) to ICT (CHO: 57.3 ± 8.3 min; CP: 57.2 ± 7.8 min) and return to baseline levels following RVT (CHO: 55.3 ± 5.3; CP: 55.7 ± 7.8 min), with no between-treatment effects. Similarly, all treatment differences in power output between CHO (NT: 191 ± 48; ICT: 188 ± 53; RVT: 198 ± 41 W) and CP (NT: 201 ± 54; ICT: 187 ± 47; RVT: 201 ± 61 W) were unclear.

#### 3.2.2. Responses during Constant-Load Exercise

**Figure 3:**
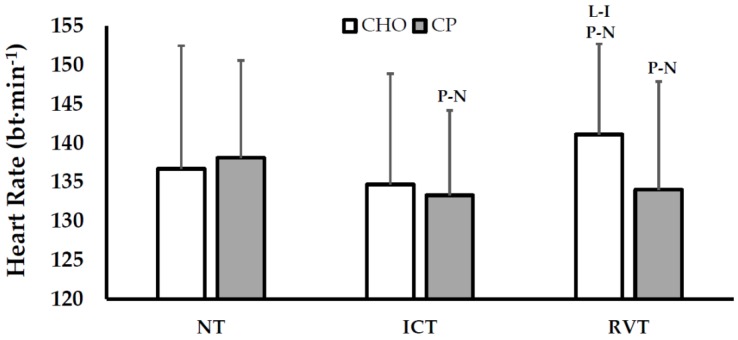
Heart Rate Responses (Mean ± SD) during Constant-Load Cycling (50% Wmax). Within-treatment effects: P-N = *Possibly* different than NT; L-I = *Likely* different than ICT. Between-treatment effects: NT-ICT = *Unclear* (% chance of larger/trivial/smaller attenuation in heart rate with CP, compared to CHO = 51/39/10), ICT-RVT = *Possible* (74/24/2), NT-RVT = *Likely* (85/12/2). NT = normal training; ICT = intensified cycle training; RVT = reduced volume training; CHO = carbohydrate supplementation; CP = carbohydrate + protein supplementation.

Physiological responses during constant-load cycling were examined at 50% W_max_, corresponding to 57%–62% VO_2peak_. Heart rate responses are shown in Figure 3. Briefly, heart rate was possibly decreased following ICT with CP, but not altered in CHO. Following RVT, heart rates possibly increased in the CHO condition (versus ICT and NT), while heart rate remained attenuated with CP (versus NT). As a result of these combined effects, reductions in heart rate between NT-RVT with CP were *likely* greater than CHO.

Constant-load exercise data for VO_2_, RER, glucose, lactate, and RPE are reported in Table 3. Treatment differences between training periods for most variables were unclear. However, RER responses exhibited differences between treatments. In general, RER tended to decrease from NT-ICT with CP, and return to baseline levels following RVT. By contrast, RER values trended upwards throughout the course of the study in the CHO condition.

#### 3.2.3. VO_2peak_ and Body Weight

VO_2peak_ possibly increased in the CHO condition from NT-ICT and from NT-RVT (NT: 4457 ± 833 mL/min; ICT: 4592 ± 736 mL/min; RVT: 4675 ± 887 mL/min) but not CP (NT: 4337 ± 973 mL/min; ICT: 4335 ± 1032 mL/min; RVT: 4433 ± 1107 mL/min). As a result, changes in VO_2peak_ from NT-ICT and NT-RVT were possibly greater with CHO versus CP (an order-adjusted analysis to account for effects of treatment order provided the same semantic inference). All treatment differences in body weight between CHO (NT: 70.7 ± 11.7; ICT: 70.1 ± 11.3; RVT: 70.6 ± 11.3 kg) and CP (NT: 70.9 ± 11.7; ICT: 71.0 ± 11.4; RVT: 70.9 ± 11.6 kg) were *most likely* trivial.

#### 3.2.4. Muscle Function, Size and Soreness

**Figure 4:**
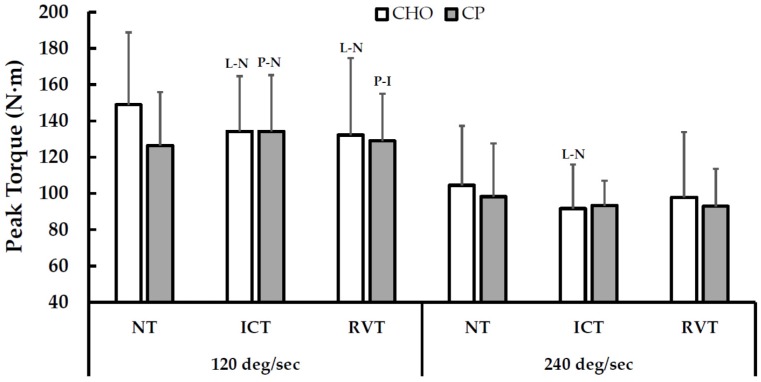
Effects of Training Periods and Nutritional Supplementation on Peak Knee Extensor Torque. Within-treatment effects: P-N = *Possibly* different than NT; L-N = *Likely* different than NT; P-I = *Possibly* different than ICT. Between-treatment effects at 120 deg/s: NT-ICT = *Likely* (% chance of smaller/trivial/larger decrease in torque with CP, compared to CHO = 95/5/1), ICT-RVT = *Unclear* (32/35/33), NT-RVT = *Unclear* (59/31/10). Between-treatment effects at 240 deg/s: NT-ICT = *Unclear* (64/25/11), ICT-RVT = *Likely* (2/14/84), NT-RVT = *Unclear* (16/29/55). NT = normal training; ICT = intensified cycle training; RVT = reduced volume training; CHO = carbohydrate supplementation; CP = carbohydrate + protein supplementation.

Peak torque values for each training period and nutritional treatment are shown in Figure 4. In general, declines in peak torque from NT to ICT tended to be greater with CHO than with CP. However, treatment differences did not persist following RVT (this interpretation is consistent with order-adjusted analyses to account for effects of treatment order from NT-RVT, as all treatment effects at that time point were unclear).

**Figure 5:**
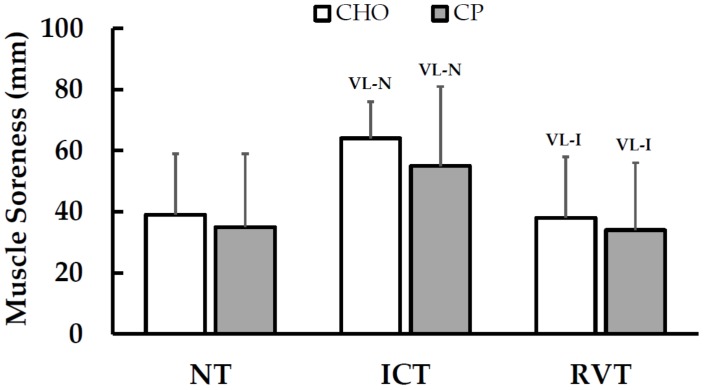
Effects of Training Periods and Nutritional Supplementation on Muscle Soreness (Mean ± SD). Within-treatment effects: VL-N = *Very likely* different than NT; VL-I = *Very likely* different than ICT; No between-treatment effects were observed. NT = normal training; ICT = intensified cycle training; RVT = reduced volume training; CHO = carbohydrate supplementation; CP = carbohydrate + protein supplementation.

Changes in muscle fiber cross-sectional area are shown in Table 4. There were no clear changes amongst fiber CSA throughout the CHO treatment. However, with CP supplementation, MHC I CSA *very likely* increased (13.6% ± 8.0%) and MHC IIa *likely* increased (16.4% ± 19.4%) from NT to ICT. This resulted in a *likely* treatment difference in MHC I fiber CSA response from NT to ICT (comparisons to the RVT time point are not available due to insufficient tissue yields for 2 subjects in CP). Muscle soreness values for each training period are shown in Figure 5. Soreness *very likely* increased from NT to ICT (with both CHO and CP) and *very likely* decreased to baseline levels following RVT, with no clear treatment differences.

#### 3.2.5. Serum Biomarkers: Albumin, Creatine Kinase, and Cortisol

Serum albumin levels tended to increase from NT to ICT to a similar extent between CHO (*likely*, 0.5 ± 0.5 g·dL^−1^) and CP (*unclear*, 0.4 ± 0.6 g·dL^−1^). There were no clear treatment effects on albumin levels. Serum albumin levels were as follows for CHO (NT: 5.73 ± 1.07; ICT: 6.23 ± 0.67; RVT: 6.05 ± 1.03 g·dL^−1^) and CP (NT: 5.48 ± 0.76; ICT: 5.91 ± 0.54; RVT: 6.03 ± 0.64 g·dL^−1^). Reported values are from 6 subjects, as one subject was unable to provide serum samples for all time points.

Creatine kinase levels possibly decreased from NT to ICT (−25 ± 28 U/L) with CHO but not PRO (1 ± 20 U/L; *unclear*), leading to *likely* treatment differences at these time points. All other treatment comparisons in creatine kinase between CHO (NT: 157 ± 82; ICT: 135 ± 61; RVT: 94 ± 42 U/L) and CP (NT: 127 ± 47; ICT: 128 ± 34; RVT: 137 ± 79 U/L) were unclear.

There were no clear differences in serum cortisol levels between any time points, and no treatment effects. Serum cortisol levels were: CHO = NT: 103 ± 37; ICT: 97 ± 32; RVT: 94 ± 42 ng/mL and CP = NT: 125 ± 47; ICT: 114 ± 26; RVT: 107 ± 33 ng/mL.

## 4. Discussion

The central objective of this study was to assess the effects of CP supplementation on time trial performance, skeletal muscle function and morphology, and heart rate responses following a prolonged period of heavy training and recovery. Cycling performance was modestly impaired following ICT (2.4% slower versus NT) and restored with RVT, with no apparent performance differences between CP and CHO. Peak isokinetic torque of the knee extensors was diminished following ICT in the CHO condition, but was better preserved with CP. Similarly, both MHC I and MHC IIa cross-sectional areas were unchanged following ICT with CHO, but were increased with CP. In addition, reductions in constant-load heart rate between NT-RVT tended to be greater with CP than CHO. Altogether it appears that CP supplementation impacted select physiological parameters during heavy training and recovery, but not on a magnitude that influenced time trial performance.

The current result that repeated days of CP supplementation had no effect on performance versus CHO is supported by previous data from our laboratory [12,13]. However, the distance runners studied by Luden et al. [12] tapered their training volumes slightly during a six-day intervention period, and the subjects of Gilson and colleagues [13] completed a 4-day period of soccer training with only minimal increases in training volume (+12% versus normal training). Consequently, we designed the current study to deliver a rigorous training stimulus in an attempt to maximize the potential value of CP supplementation. Along with elevated muscle soreness, and impaired skeletal muscle function, the 10-day ICT intervention impaired cycling time trial performance by 2.4%. This was comparable to a recent study which reported that power output during a 5-min time trial was reduced by 3.8% following a rigorous 6-day training camp in cyclists [16]. Similar to the present study, these investigators reported that supplemental protein during intensified training did not affect changes in performance versus supplemental CHO alone. By contrast, Witard and associates [15] reported that increased dietary protein intake (3.0 vs. 1.5 g/kg/day) modestly attenuated performance declines following a week of intensified training that elicited large declines in performance in the control condition (>10%). However, it should be noted that the protocols for protein supplementation during heavy training also varied considerably between these studies. Witard and colleagues [15] provided a relatively large amount of additional protein (+104 g/day) throughout the day, whereas Hansen [16] provided ~65 g of supplemental protein only during exercise, and the present study supplemented ~67 g of protein during/following exercise. Thus, based on the limited studies in this area, it could be speculated that CP supplementation may elicit performance benefits when larger amounts of protein are provided during heavy training that results in sizeable decreases in performance, while smaller amounts of supplemental protein do not appear to impact performance during heavy training that elicits only modest impairments in performance.

Discrepancies between prior studies regarding the influence of CP on performance following periods of heavy training may also be affected by dietary protein intake and the resulting protein balance. For instance, Rowlands and colleagues noted that positive effects on performance were observed when CP supplementation was compared to a relatively low-protein control diet (0.92 g/kg/day; [9]), or versus a moderate-protein diet which elicited a mildly negative protein balance (1.6 g/kg/day; [10]). Yet when CP supplementation was compared to a moderate-protein control diet which produced positive daily nitrogen balance, no effects of CP on performance were observed (1.5 g/kg/day; [14]). Protein balance status could also possibly explain the differences between our findings and the aforementioned results from Witard and colleagues [15]. It is possible that the protein intake in the control diet (described above) resulted in negative protein balance during heavy training. By contrast, the slightly higher dietary protein intake in the control condition of the present study (1.7 g/kg during ICT, 1.6 g/kg during RVT) could have been sufficient to elicit positive protein balance, minimizing the potential effects of CP on performance. This is consistent with aforesaid findings from Hansen and associates [16], who reported no effects of protein supplementation versus CHO when protein intake was controlled between groups at 1.7 g/kg/day. However, another study from this group reported that orienteers who received protein supplementation before and after exercise improved 4-kilometers run performance at the end of a one-week training camp, versus those who received a control diet containing 1.8 g/kg/day of protein [11]. However, nitrogen balance was not assessed in any of these studies, and numerous methodological differences make it difficult to directly compare results between studies (i.e., Hansen’s study of orienteers utilized a between-subject design, weight-bearing exercise mode, and two training sessions per day).

Though cycling performance was not influenced by treatment, both skeletal muscle function and morphology were favorably influenced by CP. Muscle function was *likely* affected by treatment at a contractile velocity of 120 deg·s^−1^. Following ICT, peak knee extensor torque was *possibly* enhanced with CP, but *likely* impaired with CHO. Interestingly, the treatment effect was *unclear* at 240 deg·s^−1^, despite peak torque *likely* being lower after ICT with CHO but not CP. The more apparent benefit of CP on whole muscle function at 120 deg·s^−1^ suggests that the slower contractile velocity (and higher torque output) is a more sensitive measure of peak muscle function. This notion is supported by the findings of Coutts et al. who observed clear effects of heavy training on peak isokinetic torque at 60 deg·s^−1^, but not at 300 deg·s^−1^ [34]. Regardless, this is the first evidence that peak skeletal muscle force is better preserved with CP supplementation during an extended period of heavy training. The practical advantage of this outcome is questionable though, as the treatment effect on muscle function neither persisted through RVT nor did it translate to better cycling performance. The latter may be attributable to the fact that the angular velocity during cycling exercise is much closer to 240- than 120 deg·s^−1^ [35]. The apparent benefit of CP throughout ICT on muscle function may in part be due to increased muscle fiber CSA observed with CP but not CHO. Contrary to earlier reports that fiber CSA decreases following heavy training [36,37,38], MHC I (*very likely*) and MHC IIa CSA (*likely*) increased following ICT with CP, but not CHO. The mechanisms mediating this response are beyond the scope of the current investigation, but CP may have increased muscle fiber size by augmenting protein synthesis rates in the early hours following exercise [39,40,41], resulting in a potentially greater net protein balance compared to CHO [39,40]. Because our CP treatment provided additional protein and calories, we cannot conclude definitively that the effects of CP on muscle function and morphology were the result of protein per se. However, augmented protein synthesis rates have been reported with CP versus isocaloric CHO treatments in prior studies [39]. Further research is needed to delineate how potential effects of protein intake on peak muscle function and fiber size translate to cycling specific performance.

CK levels and perceived muscle soreness together offer insight into the possible effects of muscle damage on changes in muscle function with training and nutrition. Muscle soreness increased as a result of ICT and decreased following RVT, as expected [3]. However, CP had no apparent influence on muscle soreness responses, which differs from a number of prior studies that have reported attenuated post-exercise soreness with CP ingestion [12,18,20,21]. CHO was associated with *possibly* reduced CK levels following ICT compared to CP, which is contrary to earlier findings showing CP-attenuated CK levels after heavy training [9,12,21]. However, the magnitude of all changes in CK in the present study (i.e., across time points and between treatments) were very small, suggesting that the effects of training and diet on CK were negligible.

Another purpose of this investigation was to assess the effects of CP supplementation on submaximal heart rate responses. During CP, constant-load heart rates *possibly* decreased following ICT, and remained *possibly* suppressed following RVT. By contrast, constant-load heart rates during CHO did not change to a meaningful degree following ICT, and *possibly* increased from ICT-RVT. As a result, reductions in heart rate during constant-load cycling were *likely* greater with CP than CHO between NT-RVT (−9 ± 9 bpm). This apparent effect of CP on heart rate is also supported by data obtained from the training sessions (Table 1). Despite exercising at an identical average power output (between treatments) during RVT, average heart rates during training were *possibly* lower in CP versus CHO (−4 ± 5 bpm). These observations are generally consistent with findings from Goto and colleagues, who reported that protein supplementation magnified training-induced reductions in heart rate following 5 days of aerobic training in previously untrained subjects [25]. Attenuated heart rate responses during exercise could be viewed as a positive adaptation to CP ingestion as they may reflect protein-mediated increases in plasma volume and stroke volume, suggesting possible reductions in cardiovascular and heat strain during exercise [23,24,25]. However, this interpretation should be made cautiously since plasma volume and stroke volume were not measured in the present study. Additionally, prior studies reported that increased plasma and/or stroke volume were related to increased plasma albumin levels with protein supplementation [23,24,25] and though plasma albumin levels tended to increase from NT-ICT, no differences were observed between CHO and CP. The extended training periods in the study precluded a careful examination of the time course of albumin changes, and the predicted influences of CP on plasma volume potentially confounds measurements of albumin concentration in the existing model, so it is unclear whether total albumin levels were affected by CP. Meaningful increases in plasma and/or stroke volume would also be expected to increase VO_2peak_ values, even in previously trained athletes [42]. However, there was no evidence that CP improved VO_2peak_ values versus CHO (conversely, there were *possible* increases in VO_2peak_ with CHO, but not CP). Lastly, we cannot exclude the possibility that the decreased exercise heart rates with CP were associated with overtraining-induced central nervous system dysfunction [43], rather than a positive adaptation to training. Therefore, despite potentially reduced heart rate responses during constant-load cycling, the overall effects of CP ingestion on cardiovascular adaptations following ICT/RVT training were equivocal.

## 5. Conclusions

The present study extends prior findings regarding the effects of carbohydrate and protein (CP) supplementation during periods of intensified training and recovery. We observed novel findings that CP (provided during and immediately post-exercise) was associated with: (1) favorable influences on skeletal muscle function and morphology following intensified cycling training; and (2) differences in constant-load heart rates versus supplemental carbohydrate (CHO), after intensified training and recovery. Despite these potentially beneficial outcomes, CP supplementation did not attenuate the modest decreases in time trial performance observed following intensified training. Future investigators are encouraged to examine the effects of differing doses and timing of CP administration during intensified training on performance outcomes. Multi-site collaborative studies assessing the effects of CP on performance should also be considered. This approach would allow the recruitment of large sample sizes, increasing the potential to detect small but athletically relevant effects on performance.

## Figures and Tables

**Table 1:** Training Loads (Mean ± SD) during Normal, Intensified, and Reduced-Volume Training.

Variable	NT			ICT			RVT		
	CHO	CP	CP-CHO Effects	CHO	CP	CP-CHO Effects	CHO	CP	CP-CHO Effects
Duration (min/day)	61.5 ± 8.3	63.0 ± 6.0	1.5 ± 4.1	137.1 ± 14.8	139.9 ± 16.9	2.7 ± 3.9	41.2 ± 4.9	42.2 ± 5.2	1.1 ± 1.8
			52/39/10			20/79/1			40/58/2
			Unclear			Likely trivial			Possible
Power output (W)	189 ± 31	182 ± 30	−8 ± 13	165 ± 32	167 ± 26	2 ± 6	193 ± 37	193 ± 36	0 ± 5
			7/21/72			30/66/3			4/93/3
			Unclear			Possible			Likely trivial
Heart Rate (bt·min^−1^)	150 ± 16	146 ± 14	−4 ± 5	139 ± 13	137 ± 13	−2 ± 6	150 ± 13	146 ± 11	−4 ± 5
			2/32/65			5/60/35			1/28/72
			Possible			Unclear			Possible

Effects are mean ± 90% CI, % likelihoods of positive/trivial/negative effects, and semantic inference. NT = normal training; ICT = intensified cycle training; RVT = reduced volume training; CHO = carbohydrate supplementation; CP = carbohydrate + protein supplementation.

**Table 2:** Macronutrient Intake (Mean ± SD) during Intensified Training and Reduced-Volume Training.

Dietary Macronutrients (Day)				During-Exercise		Post-Exercise		Total Macronutrient Intake (Day)		
CHO		CP	CP-CHO Effects	CHO	CP	CHO	CP	CHO	CP	CP-CHO Effects
***ICT Period***										
Carbohydrate (g)	337 ± 100	331 ± 114	−6 ± 15	112 ± 11	112 ± 11	86 ± 14	86 ± 14	535 ± 110	529 ± 122	−6 ± 15
			1/88/11							1/92/7
			Likely trivial							Likely trivial
Protein (g)	120 ± 38	118 ± 38	−3 ± 11	0 ± 0	38 ± 4	0 ± 0	29 ± 5	120 ± 38	184 ± 44	64 ± 12
			9/46/45							100/0/0
			Unclear							Most likely
Fat (g)	118 ± 29	118 ± 34	0 ± 7	0 ± 0	2 ± 0	7 ± 1	8 ± 1	124 ± 30	127 ± 35	3 ± 7
			1/97/2							13/84/3
			V.L. trivial							Likely trivial
Calories (kcal)	2948 ± 779	2899 ± 878	−49 ± 122	447 ± 46	612 ± 61	404 ± 65	532 ± 85	3799 ± 841	4043 ± 951	244 ± 122
			1/79/20							91/9/0
			Likely trivial							Likely
***RVT Period***										
Carbohydrate (g)	317 ± 89	292 ± 104	−25 ± 31	33 ± 4	33 ± 4	50 ± 9	50 ± 9	400 ± 94	376 ± 106	−25 ± 31
			3/27/70							3/27/70
			Possible							Possible
Protein (g)	112 ± 22	110 ± 21	−2 ± 10	0 ± 0	11 ± 1	0 ± 0	17 ± 3	112 ± 22	138 ± 24	26 ± 11
			18/48/35							100/0/0
			Unclear							Most likely
Fat (g)	105 ± 20	97 ± 13	−8 ± 8	0 ± 0	1 ± 0	4 ± 1	5 ± 1	108 ± 21	102 ± 14	−6 ± 8
			0/68/31							2/30/69
			Possible							Possible
Calories (kcal)	2706 ± 569	2527 ± 543	−178 ± 198	132 ± 15	180 ± 20	236 ± 43	310 ± 56	3074 ± 608	3018 ± 577	56 ± 188
			2/26/72							6/68/25
			Possible							Unclear

Effects are mean ± 90% CI, % likelihoods of positive/trivial/negative effects, and semantic inference. CHO = carbohydrate supplementation; CP = carbohydrate + protein supplementation.

**Table 3:** Physiological Responses (Mean ± SD) during Constant-Load Cycling (50% W_max_).

Variable	NT		ICT		RVT		Treatment Differences		
	CHO	CP	CHO	CP	CHO	CP	NT-ICT	ICT-RVT	NT-RVT
VO_2_ (mL·min^−1^)	2767 ± 368	2704 ± 496	2679 ± 404 ^(P-N)^	2563 ± 418 ^(L-N)^	2671 ± 389	2587 ± 501 ^(L-N)^	−53 ± 190	32 ± 204	−20 ± 275
							13/47/40	33/46/21	27/36/37
							Unclear	Unclear	Unclear
Heart Rate (bt·min^−1^)	137 ± 16	138 ± 13	135 ± 12	133 ± 14 ^(P-N)^	141 ± 15 ^(P-N)^	134 ± 13 ^(P-N)^	−3 ± 9	−6 ± 6	−9 ± 9
							10/39/51	2/24/74	2/12/85
							Unclear	Possible	Likely
RER	0.81 ± 0.04	0.83 ± 0.02	0.84 ± 0.11	0.80 ± 0.04 ^(L-N)^	0.87 ± 0.02 ^(ML-N)^	0.84 ± 0.03 ^(L-I)^	−0.05 ± 0.05	0.01 ± 0.08	−0.04 ± 0.03
							4/3/93	49/12/39	2/2/96
							Likely	Unclear	Very likely
Glucose (mg·dL^−1^)	87 ± 8	80 ± 6	93 ± 7 ^(L-N)^	83 ± 11	91 ± 5	86 ± 7 ^(L-N)^	−3 ± 16	5 ± 16	2 ± 9
							30/13/57	66/11/23	56/23/21
							Unclear	Unclear	Unclear
Lactate (mmol·L^−1^)	1.1 ± 0.5	1.0 ± 0.5	0.9 ± 0.1	1.0 ± 0.4	1.1 ± 0.6	0.9 ± 0.2	0.1 ± 0.8	−0.3 ± 0.7	−0.2 ± 0.8
							46/20/35	18/24/58	31/20/50
							Unclear	Unclear	Unclear
RPE (6–20)	12.0 ± 1.0	11.7 ± 2.3	12.3 ± 1.9	12.0 ± 2.2 ^(P-N)^	11.4 ± 2.2 ^(L-I)^	11.6 ± 2.2 ^(P-I)^	0.0 ± 1.1	0.4 ± 0.6	0.4 ± 1.3
							44/28/28	73/24/3	66/18/16
							Unclear	Possible	Unclear

Treatment effects are mean ± 90% CI, % likelihoods positive/trivial/negative effects, and semantic inference. ^P-N^ = *possibly* different than NT, ^L-N^ = *likely* different than NT, ^VL-N^ = *very likely* different than NT, ^ML-N^ = *most likely* different than NT, ^P-I^ = *possibly* different than ICT, ^L-I^ = *likely* different than ICT. NT = normal training; ICT = intensified cycle training; RVT = reduced volume training; CHO = carbohydrate supplementation; CP = carbohydrate + protein supplementation; VO_2_ = oxygen uptake; RER = respiratory exchange ratio; RPE = rating of perceived exertion.

**Table 4:** Muscle Fiber Size (Mean ± SD) following Normal, Intensified, and Reduced-Volume Training.

Variable	NT		ICT		RVT		Treatment Differences		
	CHO	CP	CHO	CP	CHO	CP	NT-ICT	ICT-RVT	NT-RVT
Peak Torque at 120 deg/s (N·m)	110 ± 29	93 ± 22	99 ± 23 ^(L-N)^	99 ± 23 ^(P-N)^	98 ± 31 ^(L-N)^	95 ± 19	17 ± 12 95/5/1 Likely	−2 ± 23 32/35/33 Unclear	14 ± 18 84/12/4 Likely positive
									_ADJ_ = 6 ± 16 59/31/10 Unclear
Peak Torque at 240 deg/s (N·m)	77 ± 24	72 ± 22	68 ± 18 ^(L-N)^	69 ± 10	72 ± 27	69 ± 15	6 ± 16 64/25/11 Unclear	−5 ± 12 10/42/48 Unclear	1 ± 18 47/29/24 Unclear
								_ADJ_ = −11 ± 11 2/14/84 Likely	_ADJ_ = −6 ± 16 16/29/55 Unclear
MHC I CSA (μm^2^)	5273 ± 1355	4823 ± 1319	5356 ± 897	5401 ± 1119 ^(VL-N)^	5925 ±1680	N/A	496 ± 856 80/16/4 Likely	N/A	N/A
MHC II CSA (μm^2^)	5605 ± 1512	5105 ± 2004	5685 ± 1457	5840 ± 1822 ^(L-N)^	6131 ± 1852	N/A	655 ± 1276 76/17/7 Unclear	N/A	N/A
Muscle Soreness (mm, 0–100)	39 ± 20	35 ± 24	65 ± 12 ^(VL-N)^	55 ± 26 ^(VL-N)^	38 ± 20 ^(VL-I)^	34 ± 22 ^(VL-I)^	−6 ± 21 52/26/22 Unclear	5 ± 20 54/19/27 Unclear	0 ± 17 61/13/26 Unclear

Treatment effects are mean ± 90% CI, % likelihoods of positive/trivial/negative effects, and semantic inference. ^P-N^ = *possibly* different than NT, ^L-N^ = *likely* different than NT, ^VL-N^ = *very likely* different than NT, ^VL-I^ = *very likely* different than ICT. _ADJ_ = values adjusted for *very likely* effects of treatment order. NT = normal training; ICT = intensified cycle training; RVT = reduced volume training; CHO = carbohydrate supplementation; CP = carbohydrate + protein supplementation.
